# Synthesis and study the controlled release of etronidazole from the new PEG/NaY and PEG/MCM-41 nanocomposites

**DOI:** 10.1186/2052-336X-12-35

**Published:** 2014-01-15

**Authors:** Mojgan Zendehdel, Giuseppe Cruciani, Forozan Sahra Kar, Abolfazl Barati

**Affiliations:** 1 Department of Chemistry, Faculty of Science, Arak University, Arak 38156-8-8349, Iran; 2 Department of Physics and Earth Sciences, University of Ferrara Via Saragat 1, Ferrara I-44100, Italy; 3 Department of Chemical Engineering, Faculty of engineering, Arak University, Arak, Iran

**Keywords:** PEG/NaY, PEG/MCM-41, Release, Metronidazole

## Abstract

Recently, hybrid materials using poly ethylene glycol and porous nanocrystals have been developed for drug release. In this study, a series of poly ethylene glycol (PEG)/NaY zeolite and PEG/MCM-41 nanocomposites get synthesized. These materials are characterized using FT-IR spectroscopy, XRD, TGA and SEM. After loading the metronidazole onto these nanocomposites, the release of Metronidazole was studied in two kinds of release fluids simulating body fluid KH_2_PO_4_-Na_2_HPO_4_ buffer (pH = 7.4) and gastric fluid (HCl aqueous solution, pH = 1.5) while controlling the time, pH values, and temperature using UV–vis. Results showed that these nanocomposites have further release related to NaY, MCM-41 and the order of release in two pH solutions was PEG/NaY > PEG/MCM-41 > NaY > MCM-41. The behavior of drug release in these nanocomposites is probably due to hydrogen bonding interactions between drug and the hydroxyl group on the composite framework.

## Introduction

Controlled and sustained release of drugs is important for patients requiring medicinal treatment around the clock. Controlled release has been used extensively in food, agriculture and pharmaceutical industries to deliver active substances such as drugs, pesticides, herbicides and fertilizers [1]. There are many groups of materials that take advantage of a controlled release system such as dendrimers [2], zeolites [3,4], polymers [5], MCM-41 [6], and organic–inorganic composite materials [7].

Although, controlled drug delivery technologies using polymers as carriers including natural or synthetic polymers represent one of the most rapidly advancing areas of science [8]. But, in recent years, there has been a constantly increasing interest in the application of porous silica or aluminosilcate materials as drug carriers for controlled drug release in order to meet the requirements for prolonged and better control of drug administration. Also, porous materials fulfill the requirements for homogenous distribution of drugs through the matrix in contrast to the conventionally used polymeric materials. Nano-sized particles with a well-defined morphology are receiving wide spread interest as advanced materials on the basis of a large surface area and unique physical and chemical properties which are different from bulk solids.

However inorganic materials (clay, zeolite and MCM-41) have important advantages such as high chemical and mechanical stability and low toxicity and porous structure that can be tailored to control the diffusion rate of an adsorbed or encapsulated drug; but these materials have some problems when using them in aqueous solutions and are less studied as carriers of drugs compared to organic materials such as polymers [9]. When these materials are used in aqueous solutions in all of the applications, an accumulation of them have been observed. Under physiological conditions, porous material dispersion is unstable due to high concentrations of salt. In recent years, some polymers such as poly ethylene glycol (PEG), showed steric effects which would help to stabilize the nanoparticles preventing them from agglomeration or dispersion in solutions [7]. PEG has been bound to silica through direct esterification of hydroxyl groups of its surface and the silica's surface silanols or through a urethane linkage in order to stabilize colloidal silica in water [10]. In some cases, poly (ethylene glycol) attaches to the surface of the silica particles by means of hydrogen bonding; in other cases the synthesis of the silica particles takes place directly in the presence of PEG rendering ester linkages (Si-O-C) [11].

In this research, as a model drug, metronidazole (MTZ) was used. MTZ is a nitroimidazole derivative, particularly used in treatment of anaerobic bacteria and Protozoa infections. MTZ is the drug of choice for treatment of amoebiasis, an infection concerning the large intestine caused by Entamoeba histolytica [12]. Metronidazole (MTZ) is frequently used in treatment of periodontal diseases since it can be used against several Gram-negative anaerobic rods, the pathogenesis of periodontitis, via inhibiting bacterial nucleic acid to synthesis [13].

In this contribution we tried to improve the controlled release of metronidazole. In the first section we reported the preparation and characterization of PEG/NaY zeolite and PEG/MCM-41 and at the second section we used them for releasing the metronidazole to achieve the best condition possible for drug release.

## Experimental

Poly (ethylene glycol) (PEG) M = 600), Cetyltrimethylammonium bromide(C_19_TMABr), potassium hydroxide (KOH), tetraethylorthosilicate(TEOS), N,N^’^ -methylen bis acrylamide (MBA), hydrogen chloride, N, N, N′, N′-tetra methyl ethylene diamine TMED), ammonium persulfate (APS), aluminum hydroxide, silica gel, sodium hydroxide, Metronidazole supplied by Merck. All of the mentioned substances were used without further purification. The Final product was characterized by X-ray Diffraction (XRD) patterns for all samples were acquired by a Bruker D8 Advance diffractometer equipped with a solid-state detector (Sol-X) set to discriminate CuKa radiation (15-130-2 h,0.02- step-scan, 10 s per step)., FT-IR (Galaxy series FT-IR 5000 spectrometer) TGA (Diamond TG/DTA Perkin Elmer). SEM (Philips, XL30).

### Synthesis of NaY and MCM-41

*2.1.* MCM-41 was prepared in our laboratory as follow by the previously mentioned method as follows: A gel composite with molar composition: 1TEOS: 0.13 C_19_TMABr: 5.4 HCl: 150 H_2_O was prepared and stirred at room temperature for 7 days. After filtering and washing with water, the catalyst was characterized using the XRD. The NaY zeolite was prepared and activated according to the procedure described before [14,15].

### Preparation of PEG/NaY zeolite and PEG/MCM-41

The best ratio for PEG/porous material achieved through testing that which ratio has the first hour ; hence, the solution of 0.25 g PEG and 0.025 g N, N- methylene Bis acryl amide was prepared. Then, 0.35 g porous material (NaY zeolite or MCM-41) after getting dispersed by sonication was added to this solution and stirred for 30 min at room temperature. We used Ammonium per sulfate (APS) to beginnthe polymerization and N, N, N′, N′-tetra methyl ethylene diamine (TMED) as initiator was used.

### Preparation 0f PEG/porous material/metronidazole

0.35 g of porous material (NaY zeolite or MCM-41) was added to a solution containing 0.30 g of metronidazole drug and acetone; then the mixture was stirred for 2 hrs and it got completely vaporized. After that, porous material/Metronidazole was added to 0.25 g PEG and 0.025 g N,N^’^-methylen bis acrylamide (MBA). The polymerization process carried out using ammonium persulfate (APS) as accelerator and N, N, N′, N′-tetra methyl ethylene diamine (TMED) as initiator.

### Metronidazole release from PEG/porous material

PEG/porous nanocomposite were suspended in 4 ml of phosphate buffer for pH = 7.4 and pH = 1.5. we drew off the buffer solution completely at preset sampling times, and replaced it with a fresh buffer solution; Then the Metronidazole solution was filtered and testing the drug concentrations in the solution was performed by an UV–VIS spectrophotometer at (landa = 274 nm for pH = 1.5 and 317 nm for pH = 7.4).

### Swelling studies

**Figure 1:**
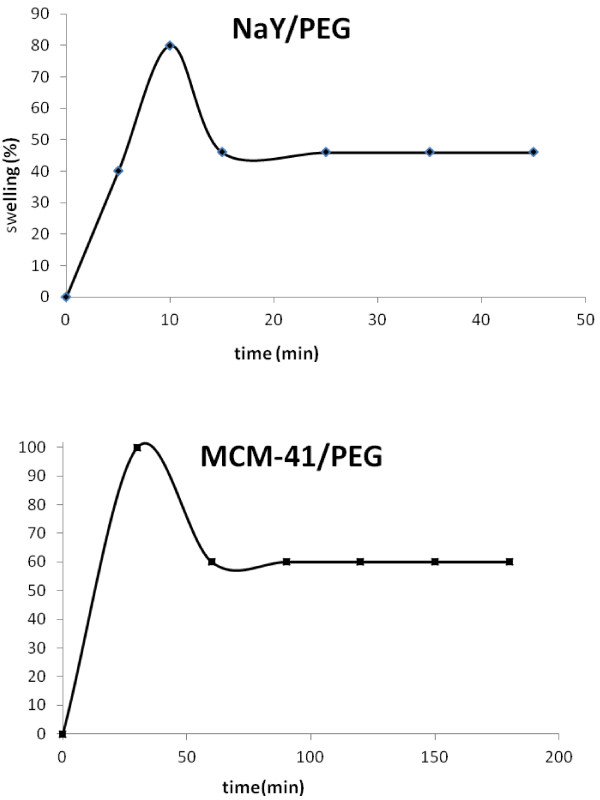
Swelling behaviour diagram of for PEG/NaY, PEG/MCM-41.

Water diffuses into the hydrogel network when it is immersed into water. This diffusion into the network is due to the osmotic pressure difference and water penetrates into the empty space between the chains. In order to study the hydrogel swelling, the dried sample is weighted, then immersed into 100 ml of distilled water that is poured into a 250 ml beaker; After a while, the sample is brought out, the excess water on its surface is dried with a tissue, and the sample is weighted again in particular time intervals. The final amount of swelling is obtained by weighing the sample after 36 hours for the last time, when no significant weight difference was observed in two successive weightings. Results of the swelling study is shown in Figure 1. Water absorption kinetics is determined by weighing different amounts of absorbed water by the gel in several time sequences. Eq. (1) is used to calculate the swelling kinetics.

(1) $$M_{t}/M_{\infty }={\mathrm{kt}}^{n}$$

Where k is the hydrogel swelling constant, n is the swelling ability, M_t_ is the amount of absorbed water at time t, and M_∞_ is the amount of absorbed water by the network at equilibrium time. The slope of the line obtained by plotting ln(Mt/M∞) versus ln(t) shows the values of n and k [16].

## Results and discussion

**Figure 2:**
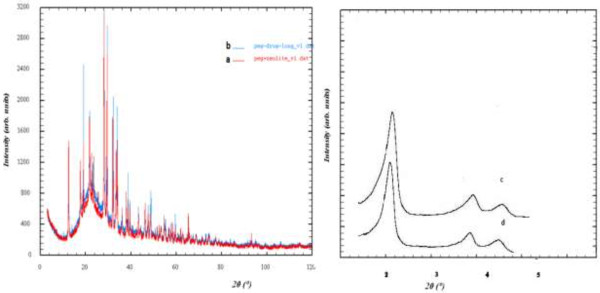
XRD pattern for PEG/NaY before (a) after (b) drug loading, PEG/MCM-41 before (c) and after (d) drug loading.

XRD patterns of PEG/NaY nanocomposite before and after the drug-loading process are shown in Figure 2a and b. In Figure 2a the NaY zeolite peaks are observable. As we can see, the structure of the zeolite does not collapse and six characteristic peaks appear at 2 of 30.1°, 35.5°, 43.1°, 53.4°, 57.0° and62.6°, which are marked by their indices ((2 2 0), (3 1 1), (4 00), (4 2 2), (5 1 1), and (4 4 0)) [17]. Figure 2b shows that the intensity of peaks decreased after Metronidazole was loaded. Powder X-ray patterns of PEG/MCM-41 nanocomposite before and after loading the drug are shown in Figure 2c and d. In Figure 2c some diffraction peaks at 2θ: 1–5 are clearly seen and can be indexed as the MCM-41 that these peak decreased after loading the mesoporous material (Figure 2d). Decrease of intensity corresponds with covering the particles with the composite [18].

**Figure 3:**
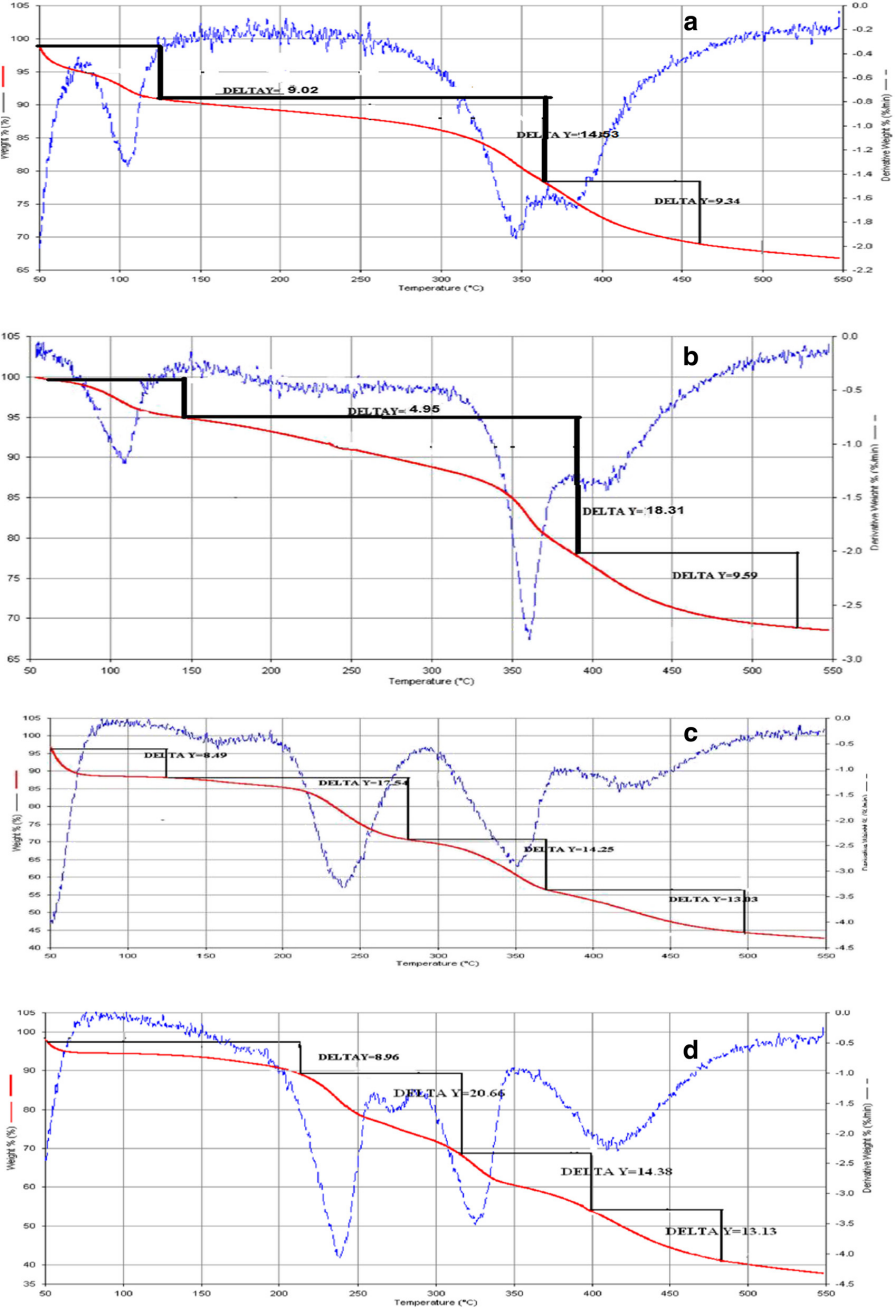
TGA/DTA for PEG/ NaY before (a) after (b) and, PEG/MCM-41 before (c) after (d) drug loading.

**Figure 4:**
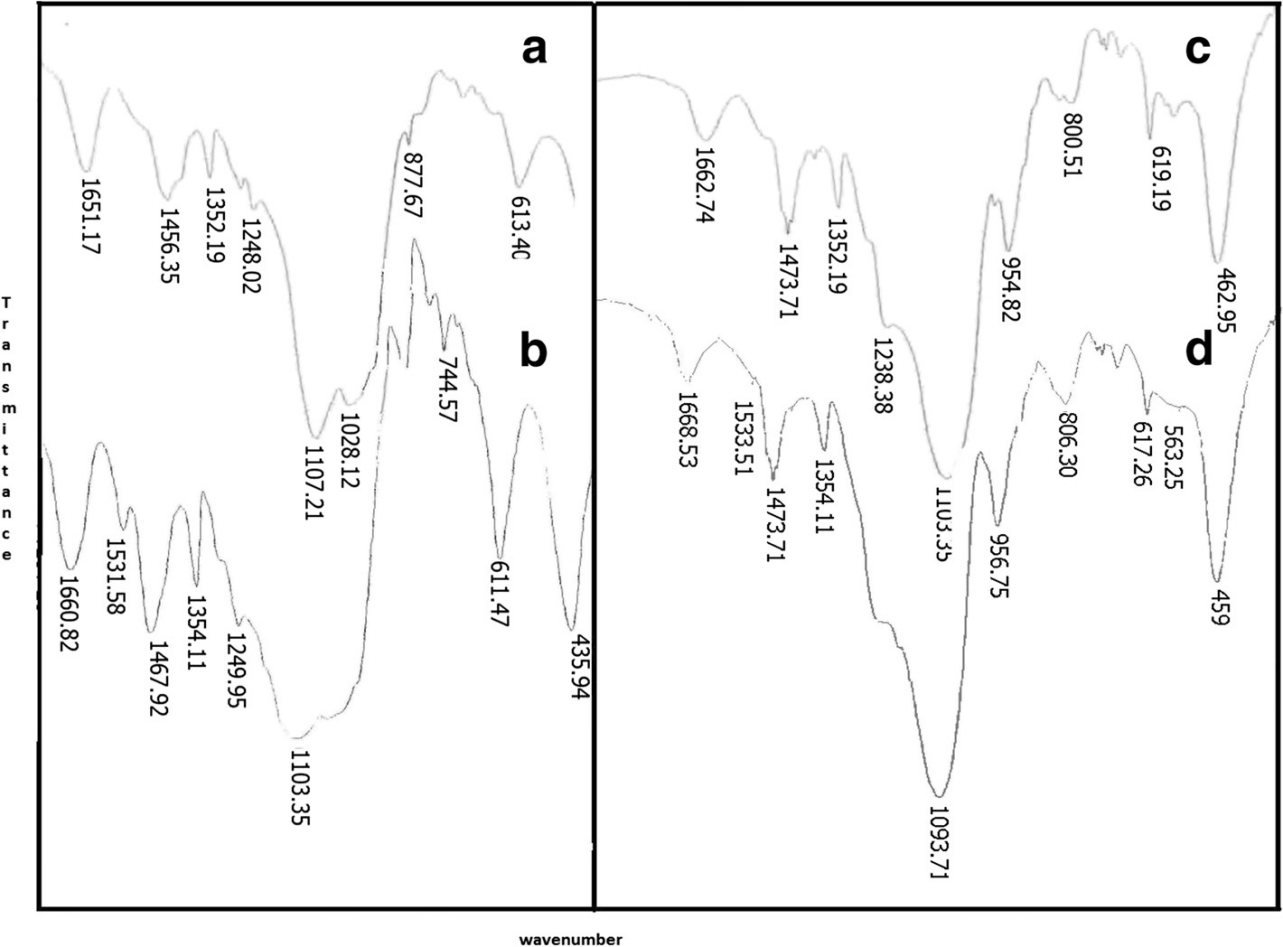
FT-IR for PEG/NaY before (a) after (b) drug loading, PEG/MCM-41 before (c) after (d) drug loading.

Figure 3a-d show TGA/DTA curves for PEG/NaY and PEG/MCM-41 nanocomposites before and after absorption of Metronidazole. Figure 3a shows three peaks regarding PEG/NaY, and the mass losses that took place at 0–110°C are related to desorbtion of water. The PEG decomposition can occur at 360°C that is about 14.03%. The last step can be related to decomposition of structure of the composite. Figure 3b shows three steps for PEG/NaY/ Metronidazole. The second step at 390°C can be assigned to the decomposition and oxidation process of the Metronidazole, which proves its presence in the composite (18.31%). The PEG/MCM-41 at Figure 3c shows four peaks that the first and second (280°C) steps are related to desorbtion of water and surfactant (8.49 and 17.54%). The PEG decomposition can occur at 370°C that is about 14.25%. The last step can be related to decomposition of structure of the composite. Figure 3d shows four steps for PEG/MCM-41/drug; the step at 310°C can be assigned to decomposition and oxidation process of Metronidazole, which proves its presence in the composite; hence, decomposition of surfactant occurs in this temperature. We saw a shift appear in all of peak to higher temperature after adding the Metronidazole. Thus, thermal stability of the composite increases when the ad micelles hosts the Metronidazole [19,20]. Figure 4a-d show FT-IR spectra in the range of 2000 to 4000 1/cm for PEG/NaY and PEG/MCM-41 nanocomposites before and after of absorption of Metronidazole. Figure 4a shows three peaks regarding hydroxyl groups of Al-OH and Si-OH in pore materials. In addition, we can see two bands at about 1070,743 cm^-1^ due to Si-O and 438 1/cm related to Al-OH in the composites. The peaks of PEG around 1352 1/cm and 3416 1/cm correspond to O-H bond and two peaks about 2876 and 1456 1/cm related to C-H bonding are shown. Figure 4c shows three peaks in regard to MCM-41 about 462,554 and 1103 1/cm. Also, the peaks related to PEG are presented around 1352 1/cm , 3416 1/cm ( O-H bond) along with two peaks about 2876 and 1456 1/cm (C-H bond). We can see a sharp peak related to NO group about 1531 1/cm and some chemical shift to lower energy for hydroxyl bond after loading the Metronidazole (Figure 4b and d).

**Figure 5:**
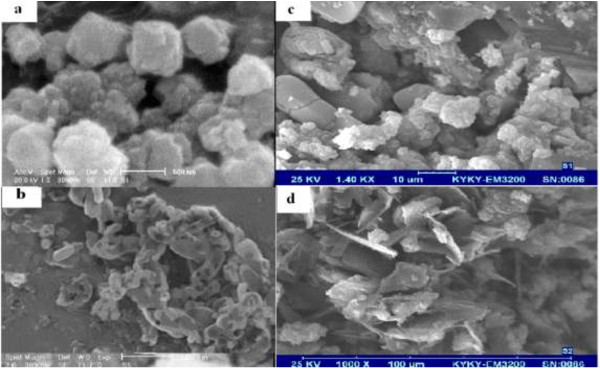
SEM forNaY (a) MCM-41 (b) PEG/NaY (c), PEG/MCM-41 (d).

SEM images of the NaY zeolite, MCM-41, PEG/NaY and nanocomposite are shown in Figure 5a-d, respectively. There is a considerable change between two samples. From the micrographs, it was found that the NaY and MCM-41 were dispersed in the polymer network. Several areas show fine network structure. It means that NaY and MCM-41 have good collaboration with PEG and improve its network which may be promising for drug adsorption [21].

### Swelling behavior

As it was mentioned before, the sample is synthesized by changing one monomer composition when the others are constant in each step. This changing in one monomer composition leads to developing an optimized composition of synthesized gel. Thus, optimized gel has a higher swelling rate in swelling experiments. The maximum amount of the swelling rate is 10 min for PEG/NaY and 30 min for PEG/MCM-41 (Figure 1a,b). For the higher time, the swelling rate decreases. The swelling ratio of composite with hydrogel depends on the hydrophilicity of the polymer chain, porous material and structure of hydrogel network [22].

## Drug release by PEG/porous materials

Release of metronidazole from NaY zeolite, MCM-41, PEG/NaY and PEG/MCM- 41 was studied in phosphate buffer solution pH = 7.4 and artificial gastric juice pH = 1.5.

**Figure 6:**
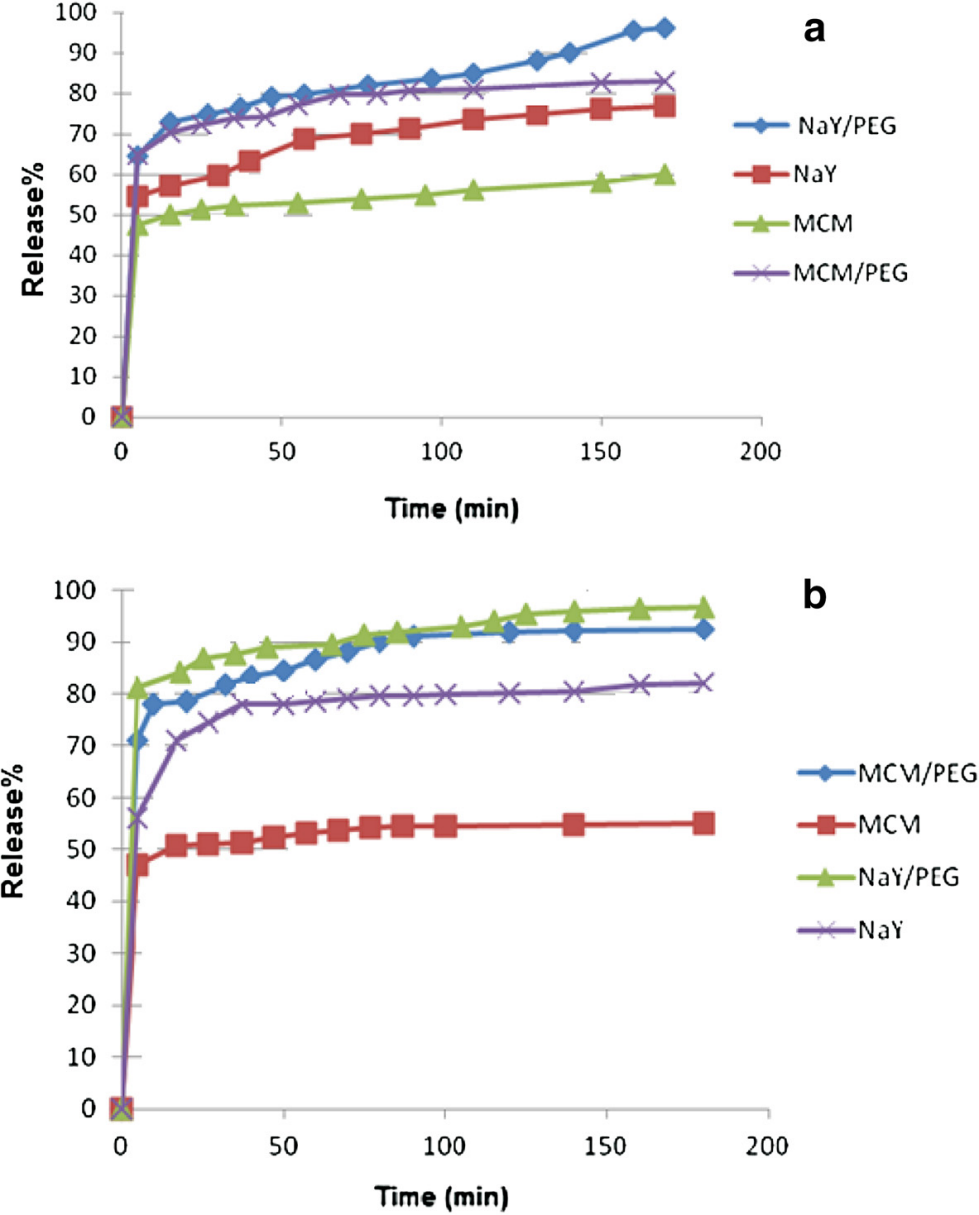
**Degree of release of the metrondazole as a functional time under mild conditions in HCl buffer (pH = 1.5) (a) and KH**_**2**_**PO**_**4**_**-Na**_**2**_**HPO**_**4 **_**buffer (pH =7.4) (b) at 37C.**

Figure 6a and b show the degree of release of metronidazole as a functional time under mild conditions in HCl buffer (pH = 1.5) and KH_2_PO_4_-Na_2_HPO_4_ buffer (pH = 7.4) at 37°C, respectively. The results show that when we used the PEG alone, all of metronidazole was released in the first minutes. The order of release of all pH for hybrid materials were as follows: PEG/NaY > PEG/MCM-41 > NaY > MCM-41. The results show that Metronidazole release is about 70 percent in the first hours which is very suitable for the fast release of drugs in mouth and stomach [23]. The fast release of drugs in mouth has attracted attention due to the advantages of such systems; because many patients (particularly pediatric and geriatric patients) find it difficult to swallow tablets and hard gelatin capsules and tend not to take their medication as prescribed. Also, there are some researches that show local delivery of metronidazole has been useful for periodontal treatment [13]. Nevertheless, about 30% of the remaining metronidazole will not release until 8 hours after taking. The good stability of these hybrids is suitable for releasing a drug within the gastro intestinal tract (e.g. targeting the stomach).

**Figure 7:**
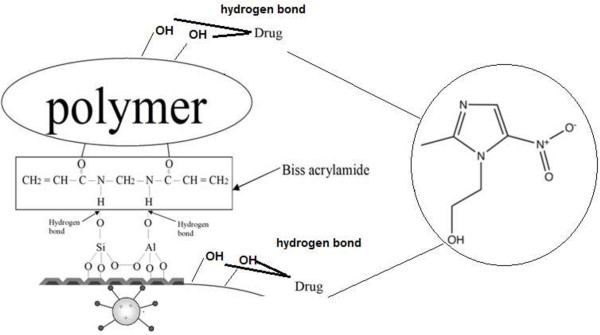
Schematic representation of formation hydrogen bond interaction and ion exchange between drug and the polymer network and the surface of zeolite, MCM-41.

The results show that the release rate for MCM-41 and PEG/MCM-41 is slower than NaY and NaY/PEG. It has been widely demonstrated that the release of drug from PEG/porous material depends on the strength and nature of the drug and composite’s chemical bonds (Figure 7). This can be explained by understanding the ability of porous materials in ion-exchange interactions. For the inclusion of metronidazole inside the channels, it seems that it’s the hydrogen bond interaction between the metronidazole molecule and the silanol groups which are present in the pore wall that causes farther keeping of the drug. It seems, NaY zeolite with Na_56_ (Al_56_ Si_136_ O_384_) formula and by almost spherical cages (12A° of diameter) tetrahedral interconnected through smaller windows (7.4A° of diameter) defined by 12 oxygen rings is not a good absorbent for metronidazole related to MCM-41 because its pore size is larger and hydrogen bond between zeolite and metronidazole is weaker [24]. Also, literature review shows that the drug release from NaY with Si/Al up to 60 is faster related to MCM-41 [4]. The results show that delivery rate of metronidazole increased when PEG was added to MCM41 and NaY. The PEG is a hydrophilic nonionic polymer that can be interacting from hydroxyl group with surface of porous material and metronidazole by hydrogen bonding. Different factors such as stability of the composite and strength of bond between the drug and the composite can affect on the rate of release. The hydrophilic groups such as hydroxyl on the PEG and porous material got hydrolyzed on the buffer solutions and it seems like the hydrogen bond between metronidazole and the composite is weaker than the porous material alone and the rate of hydrolyzed increased [7,25,26].

**Figure 8:**
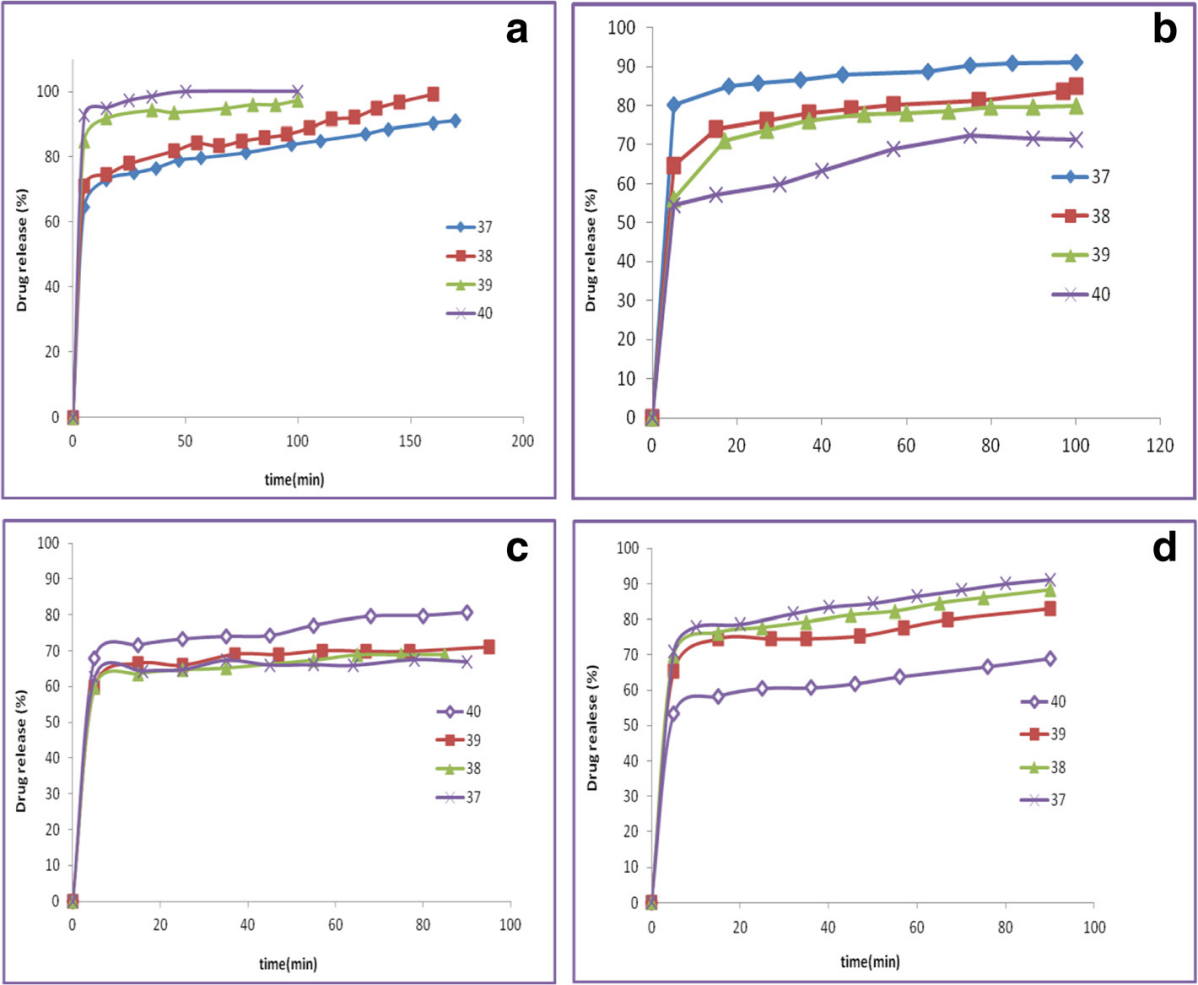
The effect of temperature on the release of metronidazole for PEG/NaY (a,b) in pH = 1.5. and pH 7.4 = PEG/MCM-41 (c,d) in pH 1.5. and pH = 7.4.

Also, Figures 8a-d show the effect of temperature on the release of metronidazole for PEG/NaY and PEG/MCM-41 in pH = 7.4 and pH = 1.5. Results show that in pH = 1.5 with increasing the temperature, release of the drug got increased and in pH = 7.4 with increasing the temperature, release of the drug was decreased. It seems when the temperature of the gel increases in acidic media, the hydrated polymer begins to lose water, leading to interactions between the side chains of the polymer and proton, especially between the hydrophobic ones, which to a large extent increases the viscosity of the gel and metronidazole bond with composite will cleavage and delivery will be done. But, in pH = 7.4 with increasing the temperature some of metronidazole that was hydrated begins to lose water and interact with the composite, hence the delivery rate decreases [27].

## Conclusion

In this work poly ethylene glycol (PEG)/NaY zeolite and PEG/MCM-41 nanocomposites were synthesized and characterized using FT-IR spectroscopy, XRD, TGA and SEM that results show good interactions between PEG and porous material. In the second step metronidazole loaded onto these nanocomposites .The release of metronidazole was studied in pH = 7.4 and HCl aqueous solution and pH = 1.5 with controlled time and temperature using UV–vis. Results showed that these nanocomposites have further release related to NaY and MCM-41 and order of release in those two pH solutions were PEG/NaY > PEG/MCM-41 > NaY > MCM-41 and about 70 percent of the realease occurred in the first hours which is very suitable for the fast release of drugs in mouth and stomach. It seems like this behavior of drug release for these nanocomposites is probably due to hydrogen bonding interactions between drug and the hydroxyl group on the composite framework.

## Competing interests

The authors declare that they have no competing interests.

## Authors’ contributions

MZ supervised the study and corresponding author. GC helped in the analysis and were AB advisors of the study. FS was the PhD investigator, designed and performed the study. All authors read and approved the final manuscript.

## References

[B1] Duncan R Seymour LW Controlled Release Technologies: A Survey of Research and Commercial Applications 1989 Oxford: Elsevier Advanced Technology

[B2] Kono K Yoshino K Takagishi T Effect of poly(ethylene glycol) grafts on temperature-sensitivity of thermosensitive polymer-modified liposomes J Control Release 2002 80 321 332 10.1016/S0168-3659(02)00018-4 11943408

[B3] Zhang H Kim Y Dutta P Controlled release of paraquat from surface-modified zeolite Y Mesopor Mater 2006 88 312 318 10.1016/j.micromeso.2005.09.026

[B4] Horcajada P Marquez-Alvarez C Ramila A Perez-Pariente J Vallet-Regi M Controlled release of Ibuprofen from dealuminated faujasitessolid state science 2006 8 1459 1465 10.1016/j.solidstatesciences.2006.07.016

[B5] Langer R Karel M Controlled release technology: polymers in medicine, food and agriculturePolym News 1981 7 6 250 258

[B6] Vallet-Regi M Ramila A Real RPD Perez-Pariente J A new property of MCM-41: drug delivery system Chem Mater 2001 13 308 311 10.1021/cm0011559

[B7] Takahashi T Yamada Y Kataoka K Nagasaki Y preparation of a novel PEG-clay hybrid as a DDS material: Dispersion stability and sustained release profiles Journal of controlled Release 2005 107 408 416 10.1016/j.jconrel.2005.03.031 16171884

[B8] Popova MD Szegedi A Kolev IN Mihály J Tzankov BS Momekov GT Lambov NG Yoncheva KP Carboxylic modified spherical mesoporous silicas аs drug delivery carriers Int J Pharm 2012 436 778 785 10.1016/j.ijpharm.2012.07.061 22884833

[B9] Gao B Fang L Men J Zhang Y Preparation of grafted microspheres CPVA-g-PSSS and studies on their drug-carrying and colon-specific drug delivery properties Mater Sci Eng C 2013 33 1300 1306 10.1016/j.msec.2012.12.029 23827575

[B10] Yague C Moros M Grazu V Arruebo M Santamar’ıa J Synthesis and steal thing study of bare and PEGylated silica micro- and nanoparticles as potential drug-delivery vectors Chem Eng J 2008 137 45 53 10.1016/j.cej.2007.07.088

[B11] Soler-Illia GJ Sanchez C Lebeau B Patarin J Chemical strategies to design textured materials: from microporous and mesoporous oxides to nanonetworks and hierarchical structures Chem Rev 2002 102 4093 4138 10.1021/cr0200062 12428985

[B12] Mura C Valenti D Floris C Sanna R Antonietta De Luca M Maria Fadda A Loy G Metronidazole prodrugs: synthesis, physicochemical properties, stability, and ex vivo release studies Eur J Med Chem 2011 46 4142 4150 10.1016/j.ejmech.2011.06.016 21726922

[B13] Pichayakorn W Prapaporn B Evaluation of cross-linked chitosan microparticles containing metronidazole for periodontitis treatment Mater Sci Eng C 2013 33 1197 1202 10.1016/j.msec.2012.12.010 23827560

[B14] Breck DW Tonawanda NY Assigned to Union Carbide, Pat. No.3130007, patented April 1964

[B15] Tanev PT Pinnavaia TJ Mesoporous silicas molecular sieves Prepared by ionic and neutral surfactant templating: a comparison of physical properties Chem Mater 1996 8 2068 10.1021/cm950549a

[B16] Buckley JD Berger M The swelling of polymer system in solvents. II. Mathematics of diffusion J Polym Sci 1962 56 175 188 10.1002/pol.1962.1205616315

[B17] Zendehdel M Barati A Alikhani H Removal of heavy metals from aqueous solution by poly(acrylamide-co-acrylic acid) modified with porous materials Polym Bull 2011 67 343 360 10.1007/s00289-011-0464-5

[B18] Xu W Gao Q Yu X Wu D Sun Y Shen Deng W Control release of ibuprofen from size-adjustable and surface hydrophobic mesoporous silica spherespowder technology 2009 191 13 20 10.1016/j.powtec.2008.09.001

[B19] Rivera A Farias T Clinoptilolite-surfactant composites as drug support: a new potential application Micropor Mesopor Mat 2005 80 337 346 10.1016/j.micromeso.2005.01.011

[B20] Lam A Rivera A Theoretical study of the interaction of surfactants and drugs with natural zeolite Micropor Mesopor Mater 2006 91 181 186 10.1016/j.micromeso.2005.11.035

[B21] Yi JZ Zhang LM Removal of methylene blue dye from aqueous solution by adsorption onto sodium humate/polyacrylamide/clay hybrid hydrogels Biores Technol 2008 99 2182 2186 10.1016/j.biortech.2007.05.028 17601732

[B22] Kim P Li N Heo SB Lee JH Novel PAAm/Laponite clay nanocomposite hydrogels with improved cationic dye adsorption behavior Compos B Eng Composites B 2008 39 756 763 10.1016/j.compositesb.2007.11.003

[B23] Pinto Joao F Site-specific drug delivery systems within the gastro-intestinal tract: from the mouth to the colon Int J Pharm 2010 395 44 52 10.1016/j.ijpharm.2010.05.003 20546856

[B24] Horcajada P Ramila A Perez-Pariente J Vallet-Regi M Influence of pore size of MCM-41 matrices on drug delivery rate Microporous Mesoporous Mater 2004 68 105 109 10.1016/j.micromeso.2003.12.012

[B25] Babazadeh M Synthesis and study of controlled release of ibuprofen from the new acrylic type polymers International J Of pharmacetutics 2006 316 68 73 10.1016/j.ijpharm.2006.02.032 16567069

[B26] Zendehdel M Barati A Alikhani H Preparation and characterization of poly(acryl amide-co-acrylic acid)/NaY and Clinoptilolite nanocomposites with improved methylene blue dye removal behavior from aqueous solution,e-polymerse-polymers 2011 2 1 13

[B27] Maderuelo C Zarzuelo A Lanao JM Critical factors in the release of drugs from sustained release hydrophilic matrices J Control Release 2011 154 2 19 10.1016/j.jconrel.2011.04.002 21497624

